# Association of corticosteroid therapy with reduced acute kidney injury and lower NET markers in severe COVID-19: an observational study

**DOI:** 10.1186/s40635-024-00670-3

**Published:** 2024-09-28

**Authors:** Sara Bülow Anderberg, Joram Huckriede, Michael Hultström, Anders Larsson, Femke de Vries, Miklos Lipcsey, Gerry A. F. Nicolaes, Robert Frithiof

**Affiliations:** 1 Department of Surgical Sciences, Anesthesiology and Intensive Care, Uppsala University, 751 85 Uppsala, Sweden; 2 Department of Biochemistry, Cardiovascular Research Institute Maastricht (CARIM), Maastricht University, Maastricht, Netherlands; 3 Department of Medical Cell Biology, Integrative Physiology, Uppsala University, Uppsala, Sweden; 4 Department of Medical Cell Biology, Uppsala University, Uppsala, Sweden; 5 Hedenstierna Laboratory, Department of Surgical Sciences, Uppsala University, Uppsala, Sweden; 6 Uppsala Centre for Paediatric Anesthesia and Intensive Care Research, Uppsala, Sweden

**Keywords:** Acute kidney injury, Corticosteroids, NETs, Histones, MPO-DNA, COVID-19

## Abstract

**Background:**

Acute kidney injury (AKI) is common in critical cases of coronavirus disease 2019 (COVID-19) and associated with worse outcome. Dysregulated neutrophil extracellular trap (NET) formation is one of several suggested pathophysiological mechanisms involved in the development of COVID-19 associated AKI. The corticosteroid dexamethasone was implemented as a standard treatment for severe COVID-19 as of June 2020. A sub-analysis of a prospective observational single center study was performed to evaluate the effect of corticosteroid treatment on AKI development and NET markers in critical cases of COVID-19.

**Results:**

Two hundred and ten adult patients admitted to intensive care at a tertiary level hospital due to respiratory failure or shock secondary to SARS-CoV-2-infection between March 13th 2020 and January 14th 2021 were included in the study. Ninety-seven of those did not receive corticosteroids. One hundred and thirteen patients were treated with corticosteroids [dexamethasone (*n* = 98) or equivalent treatment (*n* = 15)], but the incidence of AKI was assessed only in patients that received corticosteroids before any registered renal dysfunction (*n* = 63). Corticosteroids were associated with a lower incidence of AKI (19% vs 55.8%, *p* < 0.001). Fewer patients demonstrated detectable concentrations of extracellular histones in plasma when treated with corticosteroids (8.7% vs 43.1%; *p* < 0.001). Extracellular histones and in particular non-proteolyzed histones were observed more frequently with increasing AKI severity (*p* < 0.001). MPO-DNA was found in lower concentrations in patients that received corticosteroids before established renal dysfunction (*p* = 0.03) and was found in higher concentrations in patients with AKI stage 3 (*p* = 0.03). Corticosteroids did not ameliorate established AKI during the first week of treatment.

**Conclusion:**

Corticosteroid treatment in severe COVID-19 is associated with a lower incidence of AKI and reduced concentrations of NET markers in plasma.

**Supplementary Information:**

The online version contains supplementary material available at 10.1186/s40635-024-00670-3.

## Background

Acute kidney injury (AKI) is a syndrome characterized by rapidly declining glomerular filtration diagnosed by a standardized increase in serum creatinine or decline in urine output [1]. The syndrome is associated with a diverse set of underlying pathologies including infectious states such as the recent coronavirus disease 2019 (COVID-19). Renal dysfunction in critically ill COVID-19 patients is in turn common. AKI has been reported in up to 89% of cases and is associated with increased mortality [2–4]. The mechanisms leading to COVID-19-associated AKI are not fully understood but have been suggested to include endothelial dysfunction, microvascular thrombi, decreased renal perfusion, immune cell infiltration and extracellular trap formation [5–8].

Extracellular traps consist of decondensed DNA decorated with histones and enzymes such as neutrophil elastase (NE) and myeloperoxidase (MPO). The latter are found in extracellular traps originating from neutrophils so called neutrophil extracellular traps (NETs) [9]. Histones when released extracellularly due to cell injury or as a part of extracellular traps are cytotoxic to both pathogens and host cells and act as damage associated molecular patterns via pathogen recognition receptors*.*[10–12] Increasing extracellular histone concentrations have been implicated in increasing disease severity of COVID-19 [13].

Elevated histone concentrations in plasma are associated with renal dysfunction in both bacterial sepsis and community acquired pneumonia [14, 15]. Increases in NET markers such as histone-DNA, non-cleaved histone core protein 3 (H3) and MPO-DNA are linked to AKI development in COVID-19 [13, 16, 17]. Renal endothelial and epithelial cell injury, leukocyte infiltration, increased vascular permeability and local cytokine release have been described secondary to extracellular histones [18].

The Recovery trial resulted in the implementation of dexamethasone in COVID-19 patients deteriorating to respiratory support as of June 2020 as it demonstrated reduced mortality. Patients who were randomized to receive dexamethasone were also treated less frequently with renal replacement therapy, suggesting a positive effect on renal function [19].

Based on the putative inflammatory mechanism implicated in COVID-19-associated AKI and the prominent anti-inflammatory attributes of corticosteroids, we hypothesized that the administration of corticosteroids would mitigate AKI and simultaneously attenuate NET formation in critical cases of COVID-19. To evaluate this hypothesis, we conducted a sub-analysis of a prospective observational study involving critically ill patients managed both prior to and subsequent to the adoption of dexamethasone as a standard therapeutic intervention. Additionally, we sought to elucidate whether the implementation of corticosteroid therapy was associated with improvement of renal dysfunction in cases where AKI had already been established.

## Method

### Cohort

The cohort was derived from the single center prospective observational study, PRONMED, approved by the Swedish National Ethical Review Agency (EPM, Box 2110, SE-750 02 Uppsala, Sweden) (Dnr 2017-043 with amendments 2019-00169, 2020-01623, 2020-02719, 2020-05730, 2021-01469 and 2022-00526-01). The study was performed in accordance with the Declaration of Helsinki and the trial was registered a priori (Clinical Trials ID: NCT04316884). The Strengthening the Reporting of Observational Studies in Epidemiology (STROBE) guidelines were followed [20].

The study was conducted in the general intensive care unit at Uppsala University Hospital, a tertiary hospital in Uppsala, Sweden. All adult patients (> 18 years) admitted to intensive care with respiratory failure or shock due to suspected or confirmed SARS-CoV-2 infection were eligible for inclusion. Pregnancy was the only exclusion criterion. SARS-CoV-2 was diagnosed using polymerase chain reaction analysis of nasopharyngeal swabs. Informed consent was obtained from the patient or from next of kin if the patient was rendered unable because of their clinical state. In those situations where initial consent was obtained from next of kin, the patient as soon as feasible was given information and their wishes were adhered to without exception. This particular sub-study cohort was included between March 13th 2020 and January 14th 2021, including the first and second waves of the pandemic in Sweden before the advent of the Delta variant.

### Data collection

Medical history and routine laboratory analyses were extracted from electronic medical journals. Baseline characteristics were recorded upon admission. Implemented organ support in terms of vasopressor use, mechanical ventilation or continuous renal replacement therapy (CRRT) were also extracted from the electronic journals. Free days from organ support were calculated as 30 days minus the number of days of relevant treatment. The degree of respiratory failure was estimated as the ratio of arterial oxygen partial pressure (mmHg) to inspired oxygen fraction (PaO_2_/FiO_2_). AKI was diagnosed according to the Kidney Disease: Improving Global Outcome (KDIGO) creatinine and/or urine output criteria [1]. Baseline creatinine was defined as the lowest registered plasma creatinine in the year prior to ICU admission. The same plasma creatinine was used to stage any chronic kidney disease (CKD) at baseline. The highest creatinine during hospitalization, at the earliest 10 days before ICU admission, was used to estimate AKI severity. Urine output was examined during the first two weeks of intensive care.

### Routine chemistry and sample collection

Routine chemistry including neutrophil count, C-reactive protein (CRP) and plasma creatinine was performed at the hospital central laboratory. Neutrophil count was analyzed using a Sysmex XN™ instrument (Sysmex, Kobe, Japan). CRP and plasma creatinine were examined using Architect ci16200 (Abbott Laboratories, Abbott Park, IL, USA). Estimated glomerular filtration rate (eGFR) was calculated using the revised Lund-Malmö equation [21]. Urine creatinine was analyzed using IDMS calibrated enzymatic creatinine reagents from Abbott Laboratories (Abbott Park, IL, USA) on a BS380 instrument (Mindray, Shenzhen, China). Urine biomarkers were standardized by dividing them by the creatinine concentration in the same sample. This approach was used as with decreasing glomerular filtration rate, fractional excretion will tend to increase and, therefore, normalisation of spot urine markers to urine concentration becomes more important. Urinary creatinine-indexed values are the standard method to achieve this.

Plasma and urine samples used for NET marker analysis were collected adjacent to ICU admission. Histone detectability was analysed in plasma collected during intensive care. Plasma was collected in citrate vacutainers. The samples were centrifuged, separated and stored at − 80 °C. Urine was collected and stored at -80° C before analysis.

### Corticosteroid treatment

As a result of the publication of the Recovery Trial in June 2020, dexamethasone was implemented as a standard treatment for COVID-19 patients deteriorating to respiratory support [19]. The cohort could therefore be separated into patients treated with corticosteroids, 6 mg of dexamethasone daily or equivalent, and those who were not. To investigate their effect on AKI incidence, only patients who received corticosteroids at least 1 day prior to any registered renal dysfunction according to the KDIGO creatinine criteria were included in the comparison. Regarding their effect on NET formation markers, only those patients who were treated prior to blood and urine sampling were included in the treated group meanwhile those not treated with corticosteroids or that started later than the day before sampling were included in the not treated group. Lastly, to evaluate their effect on established AKI, changes in plasma creatinine were evaluated during the first week of corticosteroid treatment. The median day of symptom duration was calculated for corticosteroid treatment initiation. This was used as the starting point of the seven days in the not corticosteroid-treated group. It only included patients with established AKI when corticosteroid treatment was started and patients with AKI in the not corticosteroid-treated group. This particular analysis excluded patients who received CRRT.

### Marker analysis

#### Histone analysis in plasma

Plasma samples were analysed for the presence of histone H3 via a previously described method of semi-quantitative Western blotting using commercially available kits [22, 23]. In brief, equal volumes of tenfold diluted plasma samples were separated via SDS-PAGE gel electrophoresis (4–15%) always together with a known concentration, standard range (0.01–0.04 µg), of purified calf thymus H3 (Roche, Basel, Switzerland) and transferred to PVDF membranes (BioRad Laboratories, Hemel Hempstead, UK) using semi-dry blotting. After blocking, the membranes were incubated with primary anti-histone H3 antibody (1:10.000 o/n at 4 °C, ab1791, Abcam, Cambridge, UK) followed by a secondary biotin-conjugated IgG antibody (1:10.000 for 30 min at RT, ab97083, Abcam, Cambridge, UK) and a streptavidin–biotin/alkaline phosphatase complex (1:500 for 30 at RT, Vectastain ABC-Alkaline Phosphatase, Vector Laboratories, Burlingame, USA). Histone H3 bands were visualized by WesternBright ECL substrate (Advansta, San Jose, California, USA) using the iBright FL1500 Imaging System (Thermo Fisher Scientific, Waltham, Massachussetts, USA) and band densities were quantified by iBright Analysis Software v5.2.1 (Thermo Fisher Scientific) as compared to the known concentrations of purified H3 with a detection limit of 0.005 µg/ml (Supplement Fig. 1). Absence or presence of cleaved or non-cleaved histones was evaluated by manual inspection of bands and their respective molecular weight in the previously described Western blots. In case of two bands representing cleaved and non-cleaved histones the individual was categorized as having cleaved histones.

#### Enzyme analysis in plasma and urine (MPO, NE and MPO-DNA)

NE and MPO concentrations in plasma and urine were determined by ELISA technique using commercial kits from R&D systems (DuoSet ELISA, Bio-Techne, Minneapolis, USA) according to the manufacturer’s instructions. Dilution of plasma samples was 1:40 for MPO and 1:200 for NE in 1% BSA reagent diluent. Dilution of urine samples was 1:20 for both MPO and NE. The MPO-DNA concentration in plasma and urine was determined using the protocol as described by Kano et al. [24] In brief, using an anti-MPO capture antibody (Merck Millipore Corp, Burlington, Massachussetts, USA) in combination with the peroxidase labeled anti-DNA antibody (Roche Diagnostics, Indianapolis, Indiana, USA) to detect the complex of MPO-DNA in 1:4 diluted plasma or urine. The assay was compared to the result of plasma pooled from five septic patients (used as 100% reference) and normal pooled plasma (used as 0% reference). The result was presented as arbitrary units (AU).

#### Cell-free DNA analysis

The presence and concentration of cell-free DNA (cfDNA) were analyzed in plasma and urine samples via ALU-60 qPCR [25]. Alu elements are short stretches of 300 bp interspersed over one million times in our human genomic DNA (gDNA). In brief, samples were diluted 40-fold for plasma and tenfold for urine without a DNA isolation step. DNA concentrations were compared to a calibration range (from 1 to 300 ng/µl) using a purified and quantitated gDNA standard (TATAA Biocenter). gDNA standard was mixed in pooled citrated plasma or urine collected from 30 healthy volunteers. All measurements were performed in duplicates. The total reaction volume contained 5 µl TATAA Probe GrandMaster Mix/no ROX (TATAA Biocenter, Göteborg, Sweden), 0.5 µl TATAA Alu-60 assay probes (TATAA Biocenter), 2.5 µl H2O and 2 µl of a standard or sample, which was pipetted into a 96-well plate (Roche) and measured with a LightCycler 480 qPCR machine (Roche). The thermal cycling conditions started with a DNA-denaturation step at 95 °C for 2 min, followed by 40 cycles of denaturation at 95 °C for 5 s, annealing at 60 °C for 10 s, and extension at 60 °C for 30 s. The 2nd derivative analysis of the LightCycler 480 Software was used to calculate the CT and cfDNA concentrations of both the calibration curve and samples.

### Statistical analysis

Continuous variables were described using median with interquartile range (IQR) and categorical variables frequencies. *Χ*^2^ test was used to test differences in frequencies of categorical variables. For frequency counts < 5, Fisher’s exact test was applied. The Mann–Whitney *U* test or the Kruskal Wallis test was used to compare two or more groups, respectively. Dunn’s post hoc test was implemented, Bonferroni adjusted. Logistic regression was applied to explore the relationship between independent variables and dichotomous outcomes. A linear mixed-model was used to analyze repeated observations from the same subjects. Time and treatment were defined as fixed factors and subjects as a random effect. The outcome variable was logarithmically transformed. A two-sided *p* < 0.05 was considered significant. Cases with missing data were excluded. Statistical analysis was performed using R version 4.3.1.

## Results

### Patient demographics

**Fig. 1:**
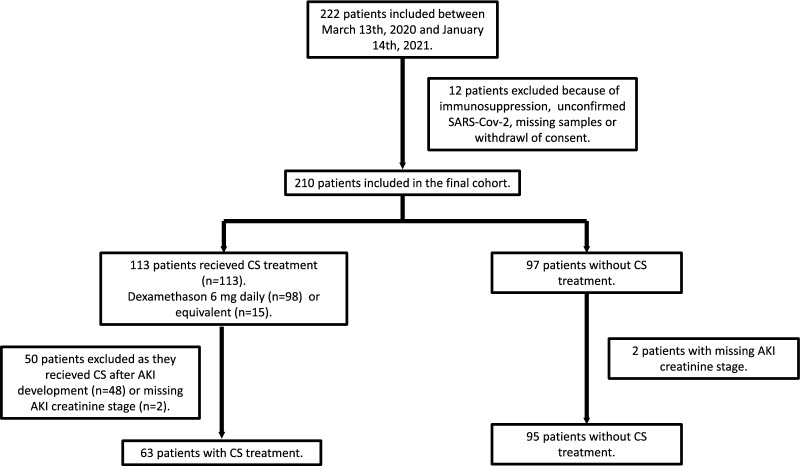
Patient groups used to investigate the effect of corticosteroid on acute kidney injury incidence. Two hundred and twenty-two adult patients were included between March 13th 2020 and January 14th 2021 due to suspected or confirmed SARS-CoV 2 infection with respiratory failure and/or shock at time of admission to intensive care. Due to unconfirmed viral infection, withdrawal of consent, renal transplant or missing plasma and urine samples 210 patients remained in the cohort. Corticosteroid (CS) treatment entailed dexamethasone 6 mg daily or equivalent treatment. Ninety-seven patients did not receive corticosteroids. Two of these patients were excluded due to missing baseline creatinine values. One hundred and thirteen patients received corticosteroids; 98 patients dexamethasone and 15 patients other forms. Only patients that had therapy initiated prior to AKI development were included in the final comparison (*n* = 63). Acute kidney injury (AKI) was diagnosed according to the KDIGO creatinine criteria exclusively

**Fig. 2:**
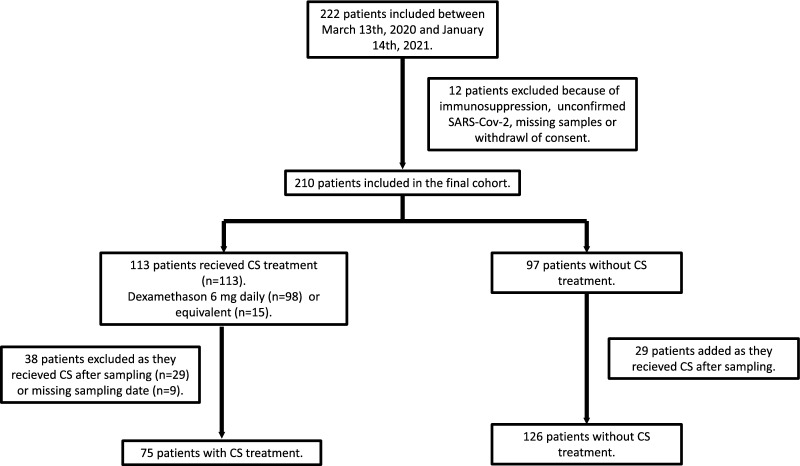
Patient groups used in investigating the effect of corticosteroids on markers of cellular damage and neutrophil activity including extracellular trap formation. Two hundred and twenty-two adult patients were included between March 13th 2020 and January 14th 2021 due to suspected or confirmed SARS-CoV 2 infection with respiratory failure and/or shock at time of admission to intensive care. Due to unconfirmed infection, withdrawal of consent, renal transplantation or missing plasma and urine samples 210 patients remained in the cohort. Corticosteroid (CS) treatment entailed dexamethasone 6 mg daily or equivalent medication. Ninety-seven patients did not receive corticosteroids. One hundred and thirteen patients received these drugs; 98 patients dexamethasone and 15 patients other forms. Twenty-nine patients were excluded due to start of corticosteroids after blood and urine sampling and an additional nine patients due to missing sampling dates

In cases of hospitalization prior to ICU admission corticosteroids were often initiated before intensive care. In the entire cohort of 210 patients, 98 patients received dexamethasone treatment. Occasionally, other drugs were administered at dosages considered equivalent to 6 mg of dexamethasone daily (*n* = 15) [26]. These therapies were mostly implemented prior to the introduction of dexamethasone as a standard treatment. The other corticosteroids used were hydrocortisone (n = 5), betamethasone (*n* = 4), methyl-prednisolone (*n* = 2) and prednisolone (*n* = 1) or these in combination (*n* = 3). To summarize, 113 patients in the cohort received corticosteroids and 97 did not. Two separate group comparisons were made. As AKI incidence was the primary outcome of interest, the first comparison was that of patients who had treatment initiated before any AKI development according to the KDIGO creatinine criteria and those who were not treated with corticosteroids (Fig. 1). The second was the effect on NET markers. Seventy-five patients had blood and urine sampled after initiation of corticosteroid treatment making a potential effect on biomarkers possible to evaluate (Fig. 2). With regard to baseline characteristics, no significant differences were observed between those treated with corticosteroids before AKI diagnosis and those who were not (Table 1). The groups were similar in terms of distribution of gender, age and body mass index as well as prevalence of hypertension, diabetes mellitus, ischemic heart disease and heart failure. There was no significant difference in eGFR at baseline. The distribution of CKD stages did not differ between the groups. During the course of intensive care, the severity of respiratory insufficiency was estimated by the PaO_2_/FiO_2_ ratio and use of mechanical ventilation. No significant differences were observed. The use of vasopressors did not differ between the treatment groups. CRRT was utilized to similar extents. There were no observed differences in 30-day or 90-day mortality.

**Table 1:** Baselines characteristics of patients not treated with corticosteroids or treated prior to acute kidney injury development during severe COVID-19

	Entire cohort	Corticosteroids		*p*
	(*n* = 210)	Yes (*n* = 63)	No (*n* = 95)	
Age (years)	64 (55–74)	63 (55–70)	60 (51–72)	0.28
Female	54 (26%)	21 (33%)	20 (21%)	0.12
BMI (kg/m^2^)	28.4 (25.4–32.9) (n = 195)	28.0 (25.3–32.3) (n = 62)	28.3 (25.5–32.9) (n = 83)	0.72
Comorbidities				
Hypertension	125 (60%)	37 (59%)	49 (52%)	0.47
Heart failure	15 (7%)	6 (10%)	4 (4%)	0.20
IHD	27 (13%)	5 (8%)	12 (13%)	0.50
Diabetes mellitus	66 (31%)	13 (21%)	28 (29%)	0.29
Baseline eGFR (ml/min/1.73m^2^)^a^	77 (65–90) (n = 192)	78 (69–90) (n = 57)	80 (69–90) (n = 84)	0.80
Baseline CKD (stage)				
1	50 (26%)	16 (28%)	27 (32%)	0.39
2	117 (61%)	37 (65%)	46 (55%)	
3	22 (11%)	4 (7%)	11 (13%)	
4	1 (1%)	0 (0%)	0 (0%)	
5	2 (1%)	0 (0%)	0 (0%)	
PaO_2_/FiO_2_^b^	80.6 (69.0–99.8) (n = 196)	87.4 (69.6–105.0) (n = 58)	81.8 (71.3–98.6) (n = 91)	0.95
Mechanical ventilation	108 (51%)	26 (41%)	51(54%)	0.17
Vasopressor	124 (59%)	29 (46%)	57(60%)	0.12
CRRT	23 (11%)	3 (5%)	9 (9%)	0.36
Free days^c^				
Intensive care	18.5 (0–24)	22 (2–25)	19 (1.5–24)	0.35
Mechanical ventilation	25 (0–30) (n = 209)	30 (14.5–30)	25 (11.5–30)	0.24
Vasopressor	27 (5.25–30) (n = 208)	30 (24–30) (n = 61)	26 (15.5–30)	0.16
CRRT	30 (0–30) (n = 209)	30 (30–30)	30 (16–30)	0.51
30-day mortality^d^	50 (24%)	12 (19%)	21 (22%)	0.79
90-day mortality^e^	57 (27%)	14 (22%)	24 (25%)	0.80

Critically ill COVID-19 patients treated with corticosteroids (CS), dexamethasone 6 mg daily or equivalent, prior to any registered renal dysfunction according to the KDIGO plasma creatinine criteria (*n* = 63) or not treated with any corticosteroid (*n* = 95). Those patients that already had an established AKI, as diagnosed by the creatinine criteria solely, at the start of steroid treatment were excluded. No significant differences were observed across treatment groups in baseline characteristics or implementation of organ support during intensive care. Group differences were explored using Mann Whitney *U* test for continuous variables and *X*^*2*^ test for categorical variables

BMI: Body mass index; IHD: Ischemic heart disease; eGFR: estimated glomerular filtration rate; CKD: Chronic Kidney Disease. CRRT: Continuous renal replacement therapy

^a^Calculated using the revised Lund-Malmö equation

^b^Arterial oxygen partial pressure (mmHg) divided by the fraction of inspired oxygen

^c^Calculated from ICU admission and the following 30 days

^d^From ICU admission

^e^From ICU admission

### Effect of corticosteroids on acute kidney injury incidence

**Fig. 3:**
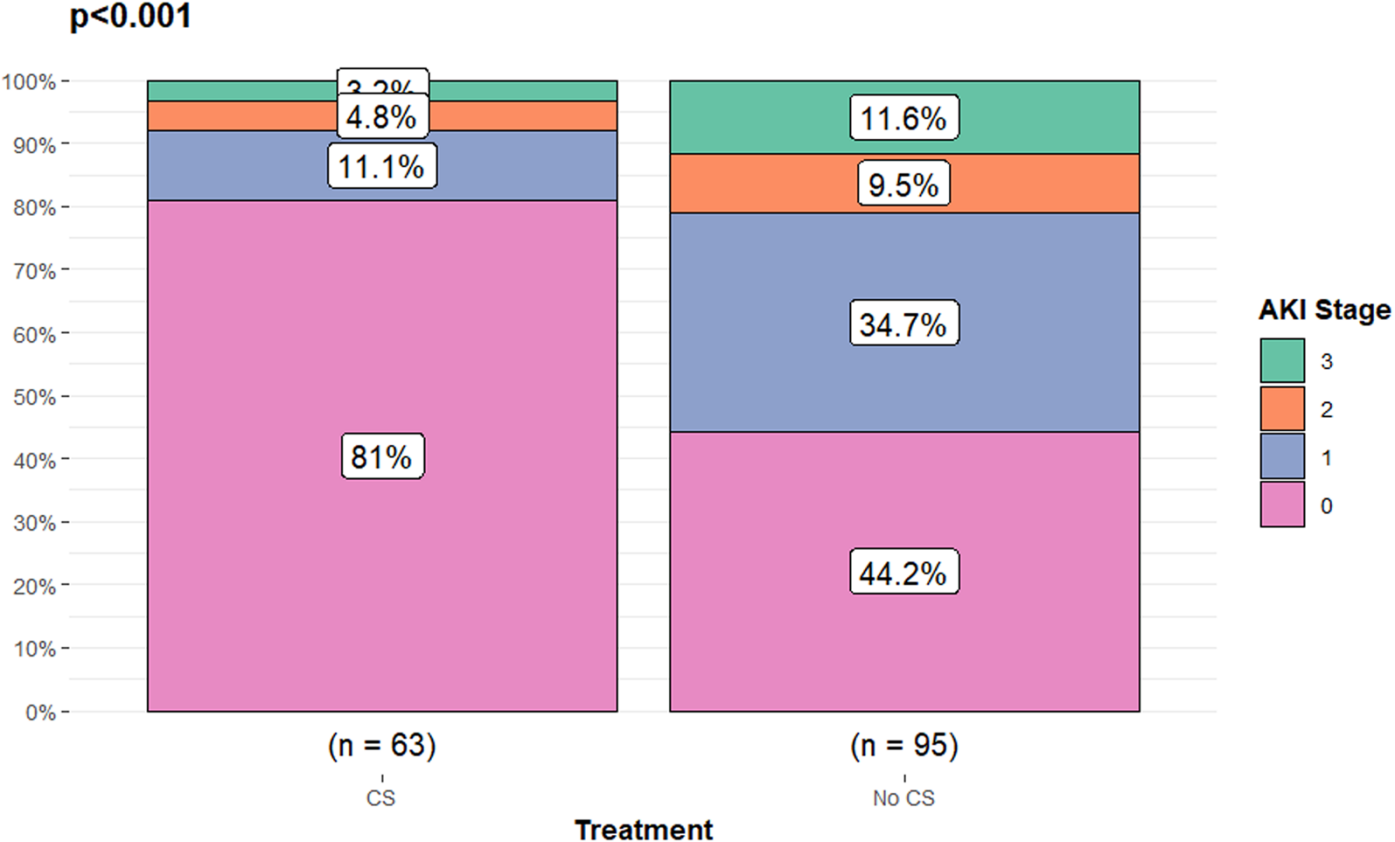
Corticosteroids and their association with acute kidney injury incidence during severe COVID-19. Acute kidney injury (AKI) was diagnosed according to the KDIGO creatinine criteria exclusively in the present analysis. Corticosteroid (CS) treatment initiated prior to any registered renal dysfunction in patients with critical COVID-19 was associated with a lower frequency of AKI development (*p* < 0.001)*.* Fifty-three (55.8%) patients not treated with corticosteroids developed AKI. In order of severity, 33 (34.7%) individuals developed stage 1, 9 (9.5%) stage 2 and 11 (11.6%) stage 3. Fifty-one (81.0%) patients did not develop AKI in the group treated with corticosteroids. The patients that did were distributed as follows; 7 (11.1%) patients reached stage 1, 3 (4.8%) stage 2 and 2 (3.2%) stage 3. Fisher’s exact test was used to compare treatment groups due to small numbers

In the entire cohort, more than half of the patients (*n* = 113 (53.8%)) developed AKI according to the creatinine criteria. Sixty-six (31.4%) patients reached stage 1, 21 (10%) stage 2, and 26 (12.4%) stage 3. When AKI was classified according to urine output, 159 (75.7%) individuals developed AKI. Twenty-nine (13.8%), 101 (48.1%) and 29 (13.8%) patients reached stages 1, 2 and 3, respectively. Corticosteroid treatment was associated with a lower AKI incidence according to the creatinine criteria. Any stage of AKI based on plasma creatinine occurred in fewer patients [*n* = 12 (19.0%)] treated with corticosteroids than in patients who were not [*n* = 53 (55.8%)] (Supplement Fig. 2). All stages of AKI were observed in the treated group, however, they were not as frequent as in patients not treated with dexamethasone or equivalent (Fig. 3). When the urine output criteria were instead implemented and corticosteroids started before a decline in urine output qualifying them for an AKI diagnosis then treatment was associated with a reduction in AKI incidence [*n* = 38 (66.7%) vs n = 80 (88.0%); *p* = 0.004] (Supplement Fig. 2). CKD was not significantly associated with AKI development according to a logistic regression model adjusted for age, gender and WHO clinical progression scale [OR (CI 95%): 1.43 (0.51–4.39)]*.*

### Histone expression, corticosteroid treatment and acute kidney injury

**Fig. 4:**
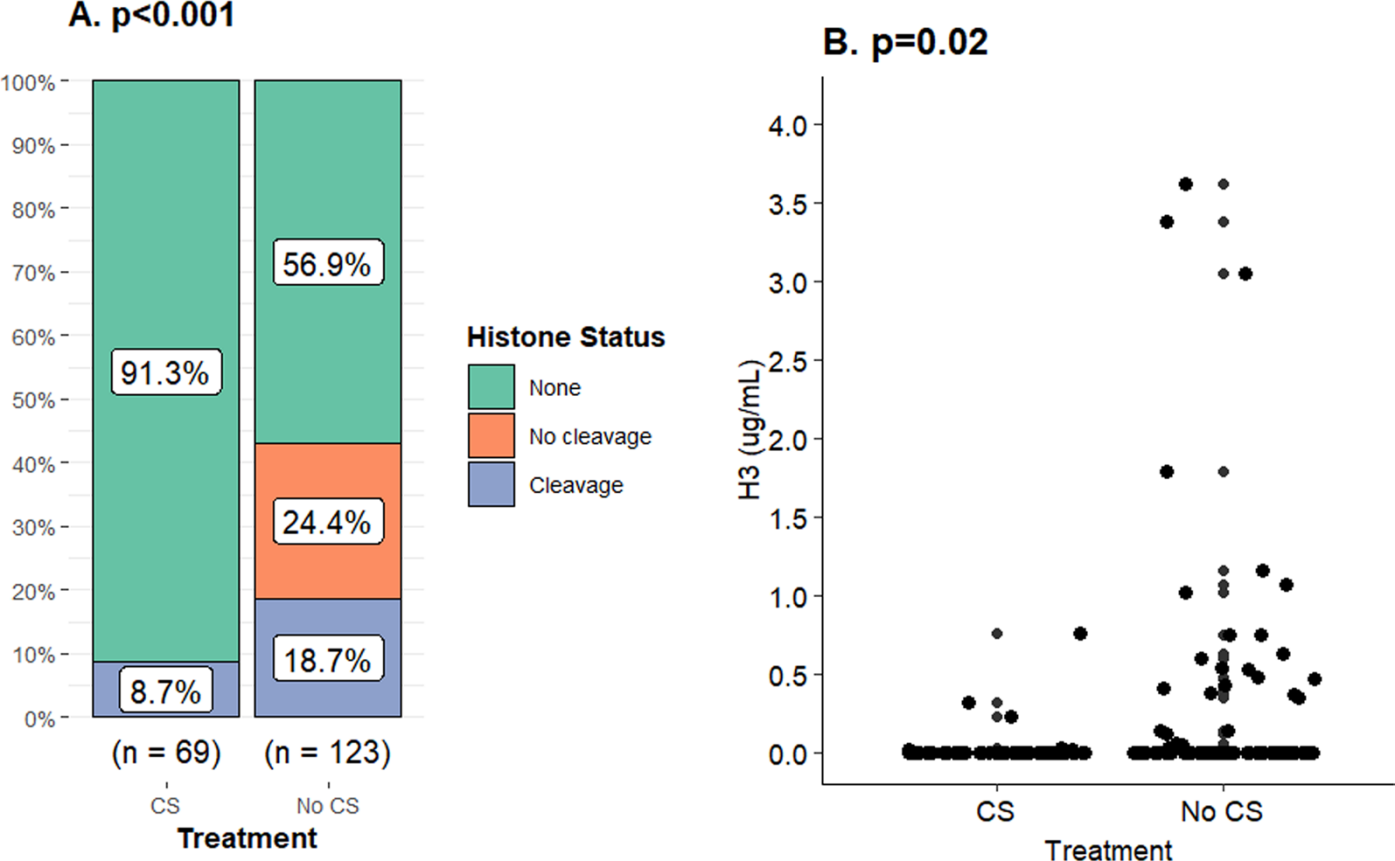
Corticosteroids and their association with extracellular histones in plasma during severe COVID-19. The effect of corticosteroids (CS), dexamethasone 6 mg daily or equivalent, on detectable extracellular histones, histone core protein 3 (H3) concentration and proteolysis of histones in plasma was evaluated in patients admitted to intensive care due to severe COVID-19. Samples were collected adjacent to admission and it was ensured that corticosteroid treatment was started prior to sampling. In this case any renal impairment was disregarded. **A** Corticosteroids were associated with reduced expression of extracellular histones in plasma [8.7% (*n* = 6) vs 43.1% (*n* = 53)] and if detected they were in the proteolyzed (cleaved) form (*p* < 0.001). Non-cleaved and cleaved histones were observed in 24.4% (*n* = 30) and 18.7% *n* = 23 of non-treated patients respectively. Fisher’s exact test was used to compare treatment groups. **B** H3 could be measured in 26 (21.1%) patients (*n* = 123) that were not treated with corticosteroids and in six (8.7%) patients (*n* = 69) that were. All individuals with H3 concentrations above 0.0 ng/ml were located within the fourth quartile in both respective treatment groups resulting in medians of 0.0 ng/ml and interquartile ranges of 0.0 ng/ml. Using the Mann Whitney *U* test, it was found that the groups were significantly different from one another. The highest observed concentrations were 3.6 µg/ml and 0.8 µg/ml, respectively (*p* = 0.02)

**Fig. 5:**
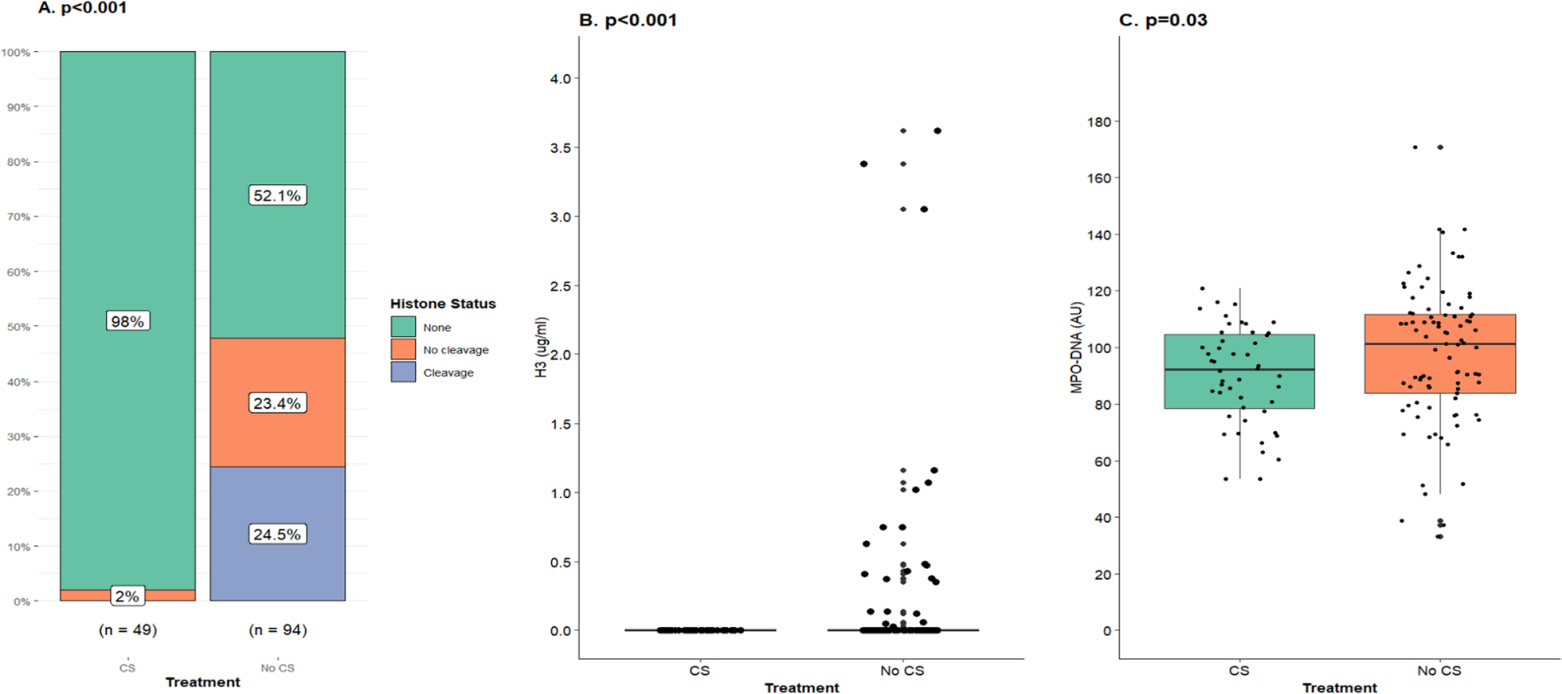
Corticosteroids implemented before acute kidney injury development and their association with extracellular histones and myeloperoxidase-DNA in plasma during severe COVID-19. The effect of corticosteroid treatment (CS), dexamethasone 6 mg daily or equivalent, was investigated on detectable extracellular histones, proteolysis of these histones and histone core protein 3 (H3) concentration in plasma in severe COVID-19 cases. Samples were collected adjacent to admission. It was ensured that corticosteroid treatment was started prior to any acute kidney injury development in the treated group (*n* = 63) of which 49 patients had plasma analyzed. Ninety-four patients not treated with corticosteroids had samples analyzed. Acute kidney injury was diagnosed according to KDIGO creatinine criteria only. **A** Treatment was associated with a reduced expression of extracellular histones in plasma [2% (*n* = 1) vs 47.9% (*n* = 45)] (*p* < 0.001) according to Fisher’s exact test. Intact (non-cleaved) and proteolyzed (cleaved) histones were observed in 23.4% (*n* = 22) and 24.5% (*n* = 23) of non-treated patients, respectively. **B** H3 concentration was estimated to 0.0 ng/ml in all of the patients treated with corticosteroids before AKI development. Twenty-two individuals had H3 concentrations above 0.0 ng/ml in the non-treated group. The groups were different according to a Mann Whitney U test using ranks. (C) MPO-DNA concentration, a marker of neutrophil extracellular trap formation, was measured in arbitrary units (AU) in plasma. The marker was observed in higher concentrations in patients without treatment (101.1 (27.7) vs 92.2 (26.0) AU) and were statistically different according to a Mann Whitney *U* test

**Fig. 6:**
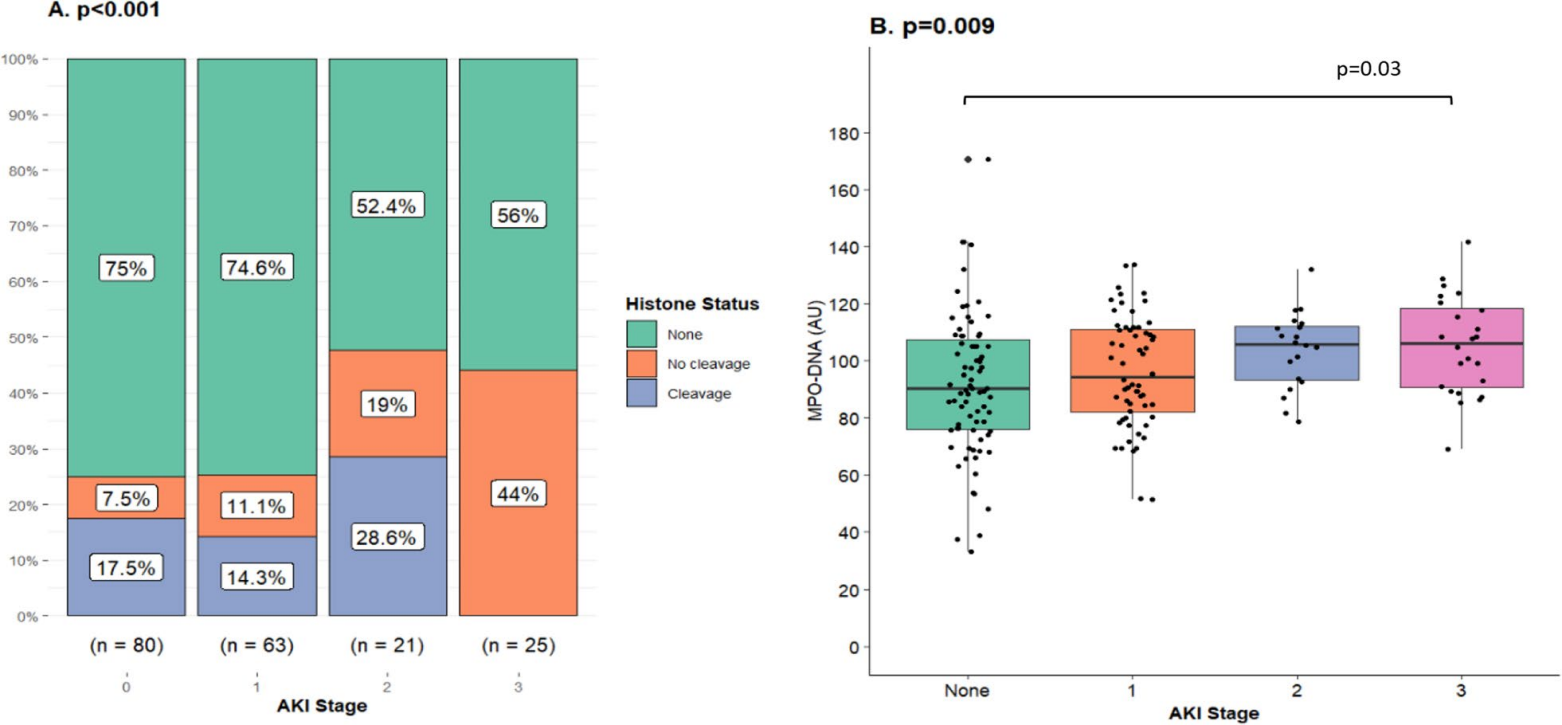
Extracellular histones and myeloperoxidase-DNA in plasma and their association with acute kidney injury severity in critical cases of COVID-19. The association between acute kidney injury stage and extracellular histones (*n* = 189) and MPO-DNA (*n* = 183) was examined. Acute kidney injury (AKI) was diagnosed according to KDIGO creatinine criteria solely. **A** Extracellular histones were detected in plasma of patients admitted to the ICU due to severe COVID-19 using a Western blot technique. If detected they were further classified as proteolyzed or not (cleaved or non-cleaved) by their molecular weight. Histones, in particular in the non-cleaved form, were associated with more severe stages of AKI (*p* < 0.001) according to a Fisher’s exact test. Extracellular histones were observed in 20 (25%) patients with no observed renal dysfunction of which the minority were non-cleaved [*n* = 6 (7.5%)]. Sixteen (25.4%) patients with AKI stage 1 had detectable histones in plasma and non-cleaved histones were observed in 7 (11.1%) patients. Nearly half of patients with AKI stage 2 [*n* = 10 (47.6%)] had detectable extracellular histones in plasma. Non-cleaved histones were found in 4 (19.0%) patients. In AKI stage 3 extracellular histones were observed in 11 (44.0%) patients and these were all in the non-cleaved form. **B** Myeloperoxidase complexed with DNA (MPO-DNA) was estimated in plasma from samples collected adjacent to ICU admission. An increase was observed across AKI stages. A Kruskal Wallis analysis indicated significant differences between groups and Dunn’s post hoc analysis demonstrated a significantly higher concentration in patients with AKI stage 3 compared to individuals with no observed renal dysfunction

Extracellular histones were classified as non-cleaved or cleaved dependent on molecular weight during Western blot. Corticosteroids were associated with less frequent detection of extracellular histones in plasma overall and in particular non-cleaved histones (Fig. 4). Treatment was also associated with lower H3 concentrations (Table 2). An additional analysis demonstrated that treatment initiated before any registered AKI development, according to the creatinine criteria, was also associated with less frequent findings of histones and non-cleaved histones in plasma in addition to lower H3 concentrations (Fig. 5 and Supplementary Table 1). The presence of extracellular histones in general and non-cleaved histones in particular in plasma was associated with more severe AKI according to the creatinine criteria when examining the whole cohort (Fig. 6). No difference across AKI stages was found in H3 concentration (*p* = 0.88). Extracellular histones and H3 analysis were attempted in urine but likely due to very low concentrations, they were not detected, and as a result not included in the results.

**Table 2:** Cell injury and neutrophil extracellular trap markers in patients treated with or without corticosteroids during severe COVID-19

Biomarker	Entire Cohort (*n* = 210)	Corticosteroids		*p*
		Yes (*n* = 75)	No (*n* = 126)	
*Plasma*				
Heparin binding protein (ng/ml)	72.8 (37.5–117.5) (*n* = 174)	74.3 (34.6–111.2) (*n* = 69)	72.1 (41.8–120.4) (*n* = 105)	0.63
Neutrophil elastase (ng/ml)	85.4 (51.2–153.8) (*n* = 192)	114.5 (67.9–169.2) (*n* = 69)	71.1 (37.6–135.2) (*n* = 122)	< 0.001
Myeloperoxidase (ng/ml)	168.6 (138.6–199.1) (*n* = 187)	184.1 (160.8–208.9) (*n* = 69)	158.8 (129.7–192.9) (*n* = 117)	< 0.001
Myeloperoxidase-DNA (AU)	95.4 (82.0–110.1) (*n* = 187)	93.3 (78.5–108.7) (*n* = 69)	99.0 (85.7–111.4) (*n* = 117)	0.22
Cell-free DNA (ng/µl)	248.7 (142.0–444.5) (*n* = 193)	241.2 (133.6–358.1) (*n* = 69)	249.2 (144.3–505.6) (*n* = 123)	0.13
Histone H3 (µg/ml)	0.0 (0.0–0.0) (*n* = 193)	0.0 (0.0–0.0) (*n* = 69)	0.0 (0.0–0.0) (*n* = 123)	0.02
*Urine*				
Cell-free DNA (ng/µl)	21.6 (4.4–86.6) (*n* = 150)	52.7 (13.7–196.4) (*n* = 56)	13.6 (1.4–59.4) (*n* = 93)	< 0.001
Neutrophil Elastase (ng/ml)	10.6 (2.8–27.9) (*n* = 170)	8.4 (3.1–20.0) (*n* = 60)	13.8 (2.3–29.5) (*n* = 109)	0.60
Myeloperoxidase (ng/ml)	17.4 (4.9–40.0) (*n* = 123)	24.5 (5.5–44.7) (*n* = 59)	15.7 (4.4–29.7) (*n* = 62)	0.37
Myeloperoxidase-DNA (AU)	49.9 (33.1–77.2) (n = 125)	49.9 (33.2–76.9) (*n* = 59)	49.7 (31.9–77.7) (*n* = 64)	0.93
*Standardised by urine creatinine*^*a*^				
Cell-free DNA [(ng/µl)/(mmol/l)]	3.7 (0.6–15.0) (*n* = 138)	10.8 (2.4–31.5) (*n* = 49)	1.8 (0.2–10.4) (*n* = 89)	< 0.001
Neutrophil elastase [(ng/ml)/(mmol/l)]	1.6 (0.3–4.4) (*n* = 155)	1.6 (0.4–4.7) (*n* = 52)	1.8 (0.2–4.0) (*n* = 103)	0.46
Myeloperoxidase [(ng/µl)/(mmol/l)]	2.3 (0.7–5.7) (*n* = 108)	2.6 (0.8–6.0) (*n* = 49)	2.1 (0.6–5.0) (*n* = 59)	0.33
Myeloperoxidase-DNA (AU/mmol/l)	7.0 (3.8–13.4) (*n* = 111)	7.7 (4.4–13.8) (*n* = 49)	5.9 (3.5–11.3) (*n* = 62)	0.26
*Routine chemistry*^*b*^				
Neutrophils (10^9^/l)	8.9 (6.4–12.2) (*n* = 138)	8.1 (6.2–12.5) (*n* = 32)	9.0 (6.6–12.1) (*n* = 104)	0.80
CRP (mg/l)	234 (154–328)	197 (140–259)	286 (188–366)	< 0.001

Critically ill patients as a result of COVID-19 infection were separated into two groups. Those treated with corticosteroids (CS), either dexamethasone 6 mg daily or equivalent (*n* = 75), before blood and urine sampling and those that were not (*n* = 126). Those patients that started treatment after blood and urine sampling were instead included in the not treated group. These groups were formed independent of AKI status. The NET markers analyzed included the enzymes neutrophil elastase (NE) and myeloperoxidas (MPO) in addition to cell free DNA (cfDNA) and extracellular histone core protein 3 (H3) concentration in plasma. In urine all markers but H3 were estimated. Urine biomarkers were also standardized by the creatinine concentration in the same sample. Group differences were explored using Mann Whitney *U* test

^a^Marker concentration divided by creatinine concentration in same sample

^b^Highest registered value during intensive care

### Myeloperoxidase-DNA, corticosteroid treatment and acute kidney injury

MPO in complex with DNA is used as a marker of NET formation [27]. The complex was observed in lower concentrations in plasma of patients that received corticosteroids before any renal dysfunction, according to the creatinine criteria, than those without corticosteroid treatment (Fig. 5 and Supplementary Table 1). The difference did not reach significance in patients that received treatment before blood sampling compared to not treated patients disregarding renal function at the time (Table 2). In patients with AKI stage 3 during hospitalization MPO-DNA was found in higher concentrations at ICU admission compared to in patients without AKI (Fig. 6).

### Corticosteroids and associated effects on neutrophil elastase, myeloperoxidase and cell free DNA

Dexamethasone or equivalent treatment were associated with higher concentrations of NE and MPO in plasma (Table 1). No significant differences in the observed enzyme concentrations were noted across AKI stages. No difference was found in plasma cfDNA concentration between the treatment groups. CfDNA was increased in the urine of corticosteroid-treated patients and when standardized by urine creatinine concentration cfDNA remained significantly increased (*p* < 0.001). No significant difference in this marker was observed across AKI stages. Changes in the urine concentrations of NE, MPO and MPO-DNA, standardized or not, did not reach significance. These markers were also compared in patients who received corticosteroids before AKI development compared to those not treated with corticosteroids (Supplementary Table 1).

### Effect of corticosteroids on established AKI

The median (IQR) elapsed time between symptom onset and start of corticosteroids was 8.5 (6 to 11) days. To investigate any ameliorating effect of corticosteroids on established AKI, patients with AKI of any stage at the start of corticosteroids (*n* = 34) were compared with patients with AKI during hospitalization who did not receive treatment (*n* = 38). Plasma creatinine during the first seven days of corticosteroid treatment was compared with creatinine values from the ninth day of COVID-19 symptoms and the following week in the non-treated group. No significant interaction was observed between time and treatment.

## Discussion

In this prospective observational study, we made two main observations. First, corticosteroids are associated with a lower incidence of AKI in patients with severe COVID-19. Secondly, they are associated with a reduced presence of histones, more specifically intact histones, and MPO-DNA in plasma.

Corticosteroids are anti-inflammatory agents that exert their effects concomitantly at multiple sites and could thereby mitigate renal injury via different potential pathways. They may diminish inflammatory damage within the lungs, consequently reducing the heightened risk of kidney injury, or attenuate the activation of the innate immune system including neutrophils in the bloodstream [28, 29]. Alternatively, corticosteroids may exert their influence locally within the kidney, suppressing the inflammatory response triggered by a systemic COVID-19 infection or a localized renal infection. The scant evidence supporting direct renal infection by SARS-CoV-2 in addition to the observed positive outcomes associated with corticosteroid treatment underscore the significance of systemic inflammation as a primary driver of renal impairment in afflicted patients [30].

Corticosteroids have previously improved glomerular filtration and tubular function in septic models which is by definition a dysregulated inflammatory state [31, 32]. The preventative use of corticosteroids in AKI associated with cardiopulmonary bypass, which induces systemic inflammation, has on the other hand rendered contradictory outcomes [33, 34]. Prior investigations in severe COVID-19 have associated dexamethasone with a positive effect on renal function. Orieux et al. performed a single center trial during nearly the same time period as ours and found a similar preventative effect of dexamethasone with regard to renal impairment [35]. An additional multicenter study associated dexamethasone with a reduced risk of severe AKI development [36]. Our results are in line with these findings. The lack of effect on established acute kidney injury in the present study may be a question of untimely and thus unbeneficial modulation of the inflammatory response. As underlined by an early trial where mortality was greatly increased in patients who received large doses of corticosteroids due to sepsis with already established renal dysfunction [37].

Neutrophil extracellular trap formation has been associated with renal impairment of various aetiologies including lupus nephritis, renal graft dysfunction and acute kidney injury secondary to ischemia, contrast administration and sepsis [12, 38–42]. NETs observed in renal micro-vessels in severe COVID-19 indicate that they may contribute to AKI development in these patients [43]. The effect of corticosteroids on NET formation and COVID-19-associated AKI combined is not extensively studied to the best of our knowledge. Their effect on NET formation in general is not fully understood. Corticosteroids have been observed to maintain, not necessarily lower, NET markers in vivo secondary to pneumonia but inhibit NET formation ex vivo [44, 45]. A small study in COVID-19 patients found no difference in NET markers secondary to dexamethasone [46]. Our finding of lower extracellular histones and MPO-DNA when treatment was implemented before deterioration in renal function may suggest an effect on neutrophil activity and/or cellular damage at that particular point in time during the disease.

Extracellular histones, released with NETs or secondary to cell damage, are proposed mediators of organ injury acting as damage associated molecular patterns and perpetuating an inflammatory response [12, 47, 48]. We observed an association between increased frequency of detectable histones and severe AKI, in line with earlier evidence that histone mediated cytotoxicity is concentration dependent [41]. The implementation of primarily dexamethasone in our cohort was paralleled by a decrease in histone detectability suggesting either reduced NET formation or ameliorated cell injury secondary to treatment. This particular marker is not specific for NETs. However, in a previous investigation, citrullinated H3, which reflects NET activity better than H3, was found in 73% of COVID-19 patients in whose plasma extracellular histones were observed [16].

NET formation is the consequence of many different triggers stimulating separate pathways in turn generating extracellular DNA scaffolds that vary in protein content. All of these steps are current areas of research. Chromatin may be decondensed through different, and overlapping, mechanisms dependent on stimuli [49]. There is protein-arginine deiminase type 4 (PAD-4) dependent unravelling of DNA via histone citrullination, neutrophil elastase in combination with myeloperoxidase dependent decondensation and additional mechanisms independent of these [50–52]. Citrullinated histones are therefore not an absolute requirement for NET formation [52–54]. MPO, a neutrophil granular enzyme which in the presence of hydrogen peroxidase oxidizes chloride, is prevalent in NET proteomic analyses [55, 56]. In visual identification of NETs the colocalization of DNA with granular agents such as MPO is recommended [54]. MPO-DNA is currently considered to be a specific and objective marker of NET formation according to a recent review [27]. It has been used repeatedly in studies evaluating systemic NET formation [43, 57–60]. However, there is not a formally established standard marker for NET formation at the present. The use of NET remnants such as both citrullinated histones and MPO-DNA have been questioned as they are subject to enzymatic breakdown. In a study of a novel assay evaluating NET capacity in sepsis ex vivo, there was a correlation between outcome and NET formation capacity but not with these more commonly used markers questioning their usefulness [61]. Taking these aspects into consideration, MPO-DNA was used as a marker of NETs in the present study. We observed higher MPO-DNA in patients with severe AKI as well as decreased in patients treated with corticosteroids before AKI development. This together indicates that the histones observed are partly of neutrophil origin and not just cellular demise.

A greater proportion of histones found in more severe cases of renal dysfunction were in a non-cleaved state. Histones independent of origin may be rendered non-injurious via two known mechanisms; neutralization through ligand binding or by proteolysis. NE, a protease, has been proposed to participate in histone proteolysis*.*[62] The observed elevation in NE concentrations during corticosteroid treatment could therefore hypothetically function as a mechanism of reducing histone-mediated cellular or renal injury. NE activity has been found to be increased many fold in severe COVID-19, however, the increase associated with corticosteroid treatment was unexpected [43].

Histones, while serving as potential markers of NET formation, may also indicate cellular injury as previously mentioned. Therefore, the observed histones could indicate general tissue damage. In turn, corticosteroids would be associated with ameliorated cell injury. The lack of a strong correlation between histone concentrations and neutrophil count may indicate a source other than NETs; however, the relationship between numbers and NET formation is not absolute. [58]

NET markers were also measured in urine, with the exception of histones. Higher cell-free DNA concentrations were associated with corticosteroids. Extracellular DNA is a proposed urine biomarker of renal injury. Urine may contain plasma derived DNA as well as DNA from renal cells and the urinary tract [63]. Our observations of an increase in urinary cell free DNA during treatment where AKI development was less frequent was, therefore, surprising. No other urinary NET marker differed between treatment groups suggesting that the DNA molecules may not originate from NETs.

### Limitations

Our study has several limitations. Its observational design, being single-center and the relatively small number of patients to start. Further studies are needed to confirm the current findings in other populations and settings. However, it was prospective, with granular data for each patient, who were followed up with repeated measurements. Another notable strength of this investigation lies in the comprehensive inclusion of the majority of COVID-19 patients admitted to the ICU at this time, characterized by a lack of patient selection. Although the time-dependent implementation of dexamethasone is a prerequisite for the present study, it comes with a selection bias. The treatment of COVID patients in intensive care changed in several ways during the early pandemic as the understanding of the disease improved. While the descriptive statistics show that the groups are similar there may be unmeasured confounders that contribute to the lower AKI incidence seen in the patients who received dexamethasone. An additional limitation is the implementation of primarily the creatinine criteria with respect to AKI development. A higher incidence of AKI was observed in our study and was described by others when the urine output criteria were used. This is often attributed in part to the conservative fluid regime as a treatment strategy for acute respiratory distress syndrome [4]. No significant relationship with histone presence was found with regard to the diuresis criteria, strengthening the argument that other mechanisms than inflammation may be of greater importance in the case of declining urine output. The creatinine criteria were also affected by multiple factors not adjusted for in this study such as fluid balance and sarcopenia. Different methods used to estimate baseline GFR have been demonstrated to be less dependable than the lowest registered plasma creatinine seven days prior to hospitalization [64]. In our case, we used the lowest registered value during the year prior to admission for COVID-19. This may result in a more accurate baseline as it was measured and not estimated [65]. We also attempted to standardize urine biomarkers with urine creatinine. No urine biomarker and, therefore, no routine for standardizing these are currently in clinical use in AKI diagnosis [66]. Alterations in GFR during non-steady states such as critical illness and developing AKI in addition to variations in tubular creatinine excretion may decrease the usefulness of this approach [67]. In addition, the method assumes an approximate linear relationship between biomarker and urine creatinine concentrations. These are novel markers in urine and we, therefore, included both absolute and standardized values. While several factors remained consistent throughout the study period, alterations in ventilation strategies and concomitant sedation may have introduced variables that could potentially influence our findings. Anesthesia affects both renal and immune function in the context of severe infections [68, 69]. Additionally, the escalated use of thrombosis prophylaxis and adjunct COVID-19 treatments over the course of the pandemic may have affected our results [70]. A few patients received corticosteroids other than dexamethasone, and although these treatments have similar biological effects, they are not identical. Their similarity in immunosuppressive effects was in this study considered more relevant and therefore included. Different variants of SARS-CoV2 may also have influenced the disease course. Finally, our omission of specific testing for citrullinated H3, which are deemed more neutrophil-specific than H3, could thus indicate both cell injury and NET formation when found.

## Conclusion

Corticosteroid treatment in critical cases of COVID-19 is associated with a lower incidence of AKI and reduced NET formation, as indicated by attenuated plasma concentrations of extracellular histones and MPO-DNA. These findings strengthen the argument that inflammatory mechanisms might mediate COVID-19 associated AKI.

## Supplementary Information

Supplementary Material 1.

Supplementary Material 2.

Supplementary Material 3.

## Data Availability

The datasets generated and/or analysed during the current study are not publicly available due to privacy and ethical restrictions but are available from the corresponding author on reasonable request.

## Abbreviations

AKI: Acute kidney injury
COVID-19: Coronavirus disease 2019
NET: Neutrophil extracellular trap
NE: Neutrophil elastase
MPO: Myeloperoxidase
DNA: Deoxyribonucleic acid
H3: Core histone protein 3
RRT: Renal replacement therapy
CS: Corticosteroids
STROBE: Strengthening the Reporting of Observational Studies in Epidemiology
CRRT: Continuous renal replacement therapy
KDIGO: Kidney Disease: Improving Global Outcome
CKD: Chronic kidney disease
ICU: Intensive care unit
CRP: C-reactive protein
eGFR: Estimated glomerular filtration rate
ELISA: Enzyme-linked immunosorbent assay
cfDNA: Cell free deoxyribonucleic acid
qPCR: Quantitative polymerase chain reaction
IQR: Interquartile range
