# Supplementary figures and images for: Association of corticosteroid therapy with reduced acute kidney injury and lower NET markers in severe COVID-19: an observational study
Source: Intensive Care Med Exp. 2024 Sep 28;12:85. doi: 10.1186/s40635-024-00670-3 (PMC11438749; doi:10.1186/s40635-024-00670-3)

M 0.01 0.02 0.04 1 2 3 4 5 6 7 8

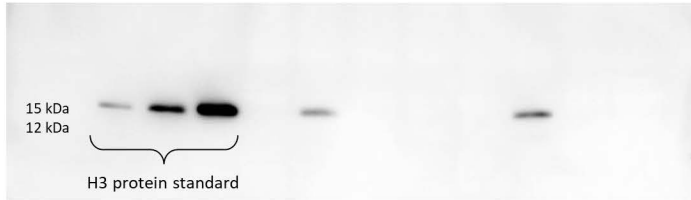

M 0.01 0.02 0.04 1 2 3 4 5 6 7 8

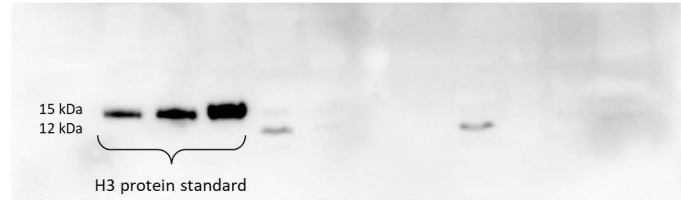

Supplement: Supplementary file 1 — Supplementary Material 1. [file 40635_2024_670_MOESM1_ESM.pdf]

**A.  $p < 0.001$**

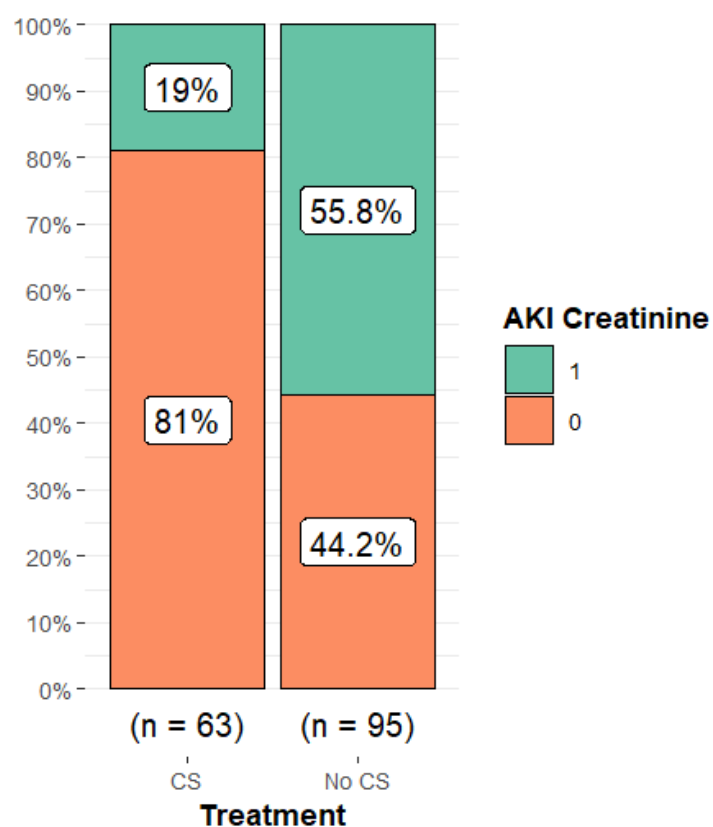

**B.  $p = 0.004$**

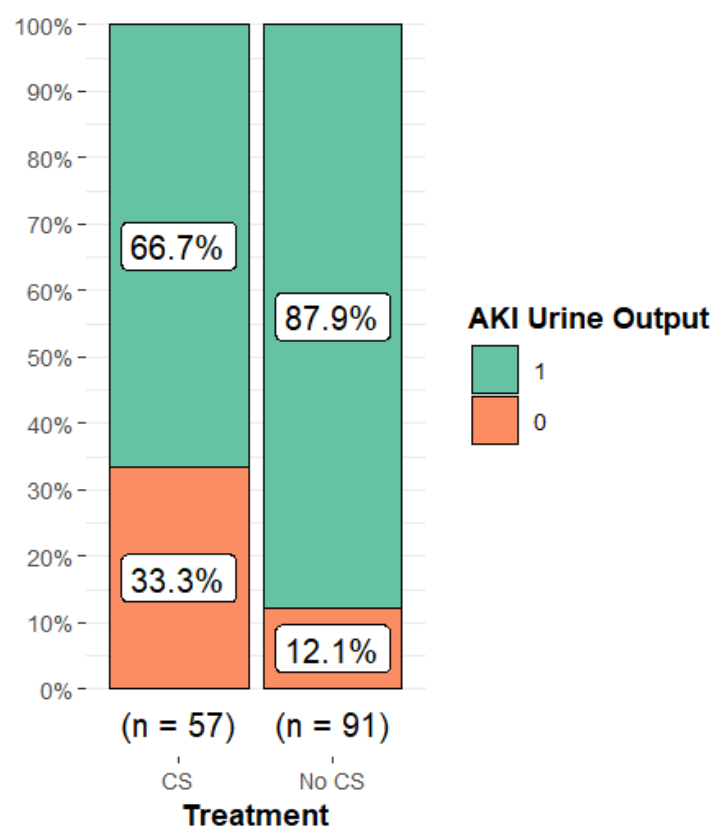

Supplement: Supplementary file 2 — Supplementary Material 2. [file 40635_2024_670_MOESM2_ESM.pdf]
