# Supplementary material for: Association of corticosteroid therapy with reduced acute kidney injury and lower NET markers in severe COVID-19: an observational study
Source: Intensive Care Med Exp. 2024 Sep 28;12:85. doi: 10.1186/s40635-024-00670-3 (PMC11438749; doi:10.1186/s40635-024-00670-3)
Supplement: Supplementary file 3 — Supplementary Material 3. [file 40635_2024_670_MOESM3_ESM.docx]

|  |  | **Corticosteroids** | |  |
| --- | --- | --- | --- | --- |
| **Biomarker** | **Entire Cohort** | **Yes (n=63)** | **No (n=95)** | **p** |
| ***Plasma*** |  |  |  |  |
| **Heparin Binding Protein (ng/ml)** | 72.8 (80.0) (n=174) | 59.5 (79.9) (n=48) | 82.3 (75.0) (n=80) | 0.04 |
| **Neutrophil Elastase (ng/ml)** | 85.4 (102.6) (n=192) | 108.2 (102.0) (n=49) | 66.7 (106.0) (n=93) | 0.01 |
| **Myeloperoxidase (ng/ml)** | 168.6 (60.5) (n=187) | 182.8 (52.6) (n=48) | 158.2 (62.8) (n=90) | 0.01 |
| **Myeloperoxidase-DNA (AU)** | 95.4 (28.1) (n=187) | 92.2 (26.0) (n=48) | 101.1 (27.7) (n=89) | 0.03 |
| **Cell-free DNA (ng/µl)** | 248.7 (302.6) (n=193) | 192.9 (236.6) (n=49) | 283.9 (394.4) (n=94) | <0.01 |
| **Histone H3 (µg/µl)** | 0 (0) (n=193) | 0 (0) (n=49) | 0 (0) (n=94) | < 0.001 |
|  |  |  |  |  |
|  |  |  |  |  |
| ***Urine*** |  |  |  |  |
| **Cell-free DNA (ng/µl)** | 21.6 (82.2) (n=150) | 32.7 (55.0) (n=43) | 5.8 (47.0) (n=67) | <0.01 |
| **Neutrophil Elastase (ng/ml)** | 10.6 (25.1) (n=170) | 6.0 (15.9) (n=49) | 13.8 (29.3) (n=81) | 0.45 |
| **Myeloperoxidase (ng/ml)** | 17.4 (35.1) (n=123) | 11.0 (36.7) (n=43) | 14.8 (20.3) (n=43) | 0.90 |
| **Myeloperoxidase-DNA (AU)** | 49.9 (44.1) (n=125) | 63.2 (45.4) (n=42) | 49.6 (42.2) (n=45) | 0.05 |
|  |  |  |  |  |
| ***Standardised by Urine Creatinine^a^*** |  |  |  |  |
| **Cell-free DNA (ng/µl/mmol/l)** | 3.7 (14.5) (n=138) | 6.3 (17.8) (n=37) | 0.6 (8.9) (n=64) | < 0.01 |
| **Neutrophil Elastase (ng/ml/mmol/l)** | 1.6 (4.0) (n=155) | 1.1 (4.6) (n=41) | 1.6 (3.4) (n=77) | 0.66 |
| **Myeloperoxidase (ng/ml/mmol/l)** | 2.3 (5.0) (n=108) | 2.2 (5.4) (n=34) | 1.3 (4.1) (n=42) | 0.58 |
| **Myeloperoxidase-DNA (AU/mmol/l)** | 7.0 (9.6) (n=125) | 8.3 (10.5) (n=34) | 5.8 (6.7) (n=44) | 0.06 |
|  |  |  |  |  |
|  |  |  |  |  |
| ***Routine Chemistry^b^*** |  |  |  |  |
| **Neutrophils (10^9^/l)** | 8.9 (5.8) (n=138) | 8.0 (6.4) (n=28) | 9.0 (5.0) (n=83) | 0.36 |
| **CRP (mg/l)** | 234 (174) (n=210) | 202 (124) (n=63) | 279 (173 ) (n=95) | <0.01 |
| ^a^Marker concentration divided by creatinine concentration in same sample. ^b^Highest registered value during intensive care. | | | | |

**Supplement Table 1.**

**Supplement Table 1. Cell damage and neutrophil extracellular trap markers in patients treated with corticosteroids prior to any registered renal impairment during severe COVID-19.**

Critically ill patients as a result of COVID-19 infection were separated into two groups. Those who started corticosteroid (CS) treatment, either dexamethasone 6 mg daily or equivalent, before any observed renal injury as diagnosed by KDIGOs acute kidney injury plasma creatinine criteria (n=63) and those that were not treated with corticosteroids (n=95). The NET markers analyzed included the enzymes neutrophil elastase (NE), myeloperoxidase (MPO) on its own and in complex with DNA (MPO-DNA), cell free DNA (cfDNA) and extracellular histone core protein 3 (H3) concentration in plasma. In urine all markers but H3 were estimated. Urine biomarkers were also standardized by the creatinine concentration in the same sample. Mann Whitney U test was applied to investigate group differences.

**Supplement Fig. 1. Detection of histone core protein 3 in the plasma of severe COVID-19 patients with Western blot.**

A molecular weight ladder was added on each blot of the 12-lane precast gels and three concentrations of histone core protein 3 (H3) in plasma. Next, 10 plasma samples were loaded and the procedure followed as described in the methods. The blot on the left shows H3 (15 kDa) in lane 2 and 7. The blot on the right shows cleaved histone H3 (12 kDa) in lanes 1 and 5. The protein standards were used to create a reference line, which was used to calculate the concentration of the H3.

**Supplement Fig. 2. Effect of corticosteroids on incidence of acute kidney injury of any severity according to plasma creatinine and urine output criteria respectively.**

Corticosteroid (CS) treatment, dexamethasone 6 mg daily or equivalent, was initiated prior to acute kidney injury (AKI) development in critically ill COVID-19 patients. (A). The incidence of AKI according to the KDIGO creatinine criteria differed significantly between those treated with corticosteroids (n=63) and those that were not (n=95, p*<*0.001). AKI was observed in 12 (19.0%) patients treated with corticosteroids compared to in 53 (55.8%) patients not treated with these drugs. (B) When using the KDIGO urine output criteria alone for AKI diagnosis, the incidence was larger in both groups than when the creatinine criteria were implemented. Thirty-eight (66.7%) patients who started corticosteroid treatment prior to any registered renal impairment according to the diuresis criteria (n=57) had a sufficiently large decline in urine output to qualify them for an AKI diagnosis during the first two weeks of intensive care. Eighty (87.9%) patients who were not treated with corticosteroids (n=91) developed AKI of some stage according to urine output. Significantly fewer patients treated with corticosteroids developed AKI *(*p*=*0.004).
